# Clinically relevant effect of rupatadine 20 mg and 10 mg in seasonal allergic rhinitis: a pooled responder analysis

**DOI:** 10.1186/s13601-019-0293-4

**Published:** 2019-10-09

**Authors:** Joaquim Mullol, Iñaki Izquierdo, Kimihiro Okubo, Giorgio Walter Canonica, Jean Bousquet, Antonio Valero

**Affiliations:** 1 Unitat de Rinologia, & Clínica de l’Olfacte, ENT Department, Hospital Clínic de Barcelona, C/Villarroel, 170, 08036 Barcelona, Catalonia Spain; 2 Institut d’Investigacions Biomèdiques August Pi i Sunyer (IDIBAPS), Barcelona, Spain; 3 Universitat de Barcelona, Barcelona, Spain; 4 CIBER de Enfermedades Respiratorias (CIBERES), Barcelona, Catalonia Spain; 5 Department of Clinical Development & Medical Adviser, Biohorm, Grupo Uriach, Avinguda Camí Reial, 51-57, 08184 Barcelona, Catalonia Spain; 6 Department of Otolaryngology-Head and Neck Surgery, Nippon Medical School, Tokyo, Japan; 7 Personalized Medicine Clinic Asthma & Allergy, Humanitas University, Humanitas Research Hospital, Rozzano, Milan, Italy; 8 MACVIA-France, Contre les Maladies Chroniques Pour un VIeillissement Actif en France European Innovation Partnership on Active and Healthy Ageing Reference Site, Montpellier, France; 9 Allergy Section, Pneumology and Allergy Department, Hospital Clínic de Barcelona, Barcelona, Spain

**Keywords:** Rupatadine, Seasonal allergic rhinitis, Responder analysis, Nasal symptoms, Ocular symptoms, Meaningful response

## Abstract

**Background:**

Different clinical trials showed the superior efficacy of rupatadine compared to placebo at improving seasonal allergic rhinitis (SAR) symptoms, but no study has assessed if the response promoted is clinically meaningful.

**Methods:**

This study is a pooled analysis of data of seven randomized, double-blind, placebo-controlled SAR studies comparing responder proportions upon treatment with rupatadine (10 or 20 mg) or placebo. We evaluated the following symptom scores at baseline (Visit 1) and over 14 days of treatment: Total 4 Nasal Symptom Score (T4NSS), Total 2 Ocular Symptom Score (T2OSS) and Total 6 Symptom Score (T6SS). The proportion of responders (50% and 75% response) and the time to response were compared between groups on days 7 (Visit 2) and 14 (Visit 3). Responder rates were compared between groups on days 7 and 14 for the complete/near-to-complete response for T4NSS (TN4SS score ≤ 2 and each symptom score ≤ 1) and T6SS (T6SS score ≤ 3 and each symptom score ≤ 1).

**Results:**

Data from 1470 patients were analyzed: 332 treated with placebo, 662 with rupatadine 10 mg and 476 with rupatadine 20 mg. The reduction in T4NSS, T2OSS and T6SS over 14 days of treatment relative to baseline was statistically higher in rupatadine groups vs the placebo group, with greater improvements in the 20 mg group. A statistically higher proportion of patients reached the 50% and 75% response for T4NSS, T2OSS and T6SS in rupatadine groups compared to the placebo group across the visits. Among rupatadine-treated patients, those receiving 20 mg compared favourably for both cut-off responses. The time to achieve a proportion of responders was shorter in the rupatadine 20 mg group than in the rupatadine 10 mg and placebo groups for all the symptom scores. The number of patients who achieved a complete/near-to-complete response for both symptom scores was higher in rupatadine groups than in the placebo group, with higher proportions in the 20 mg group.

**Conclusions:**

This responder analysis confirms the superior efficacy of rupatadine vs placebo to treat SAR. Rupatadine promoted higher proportions of responders according to stringent response criteria and in a dose-dependent manner, with faster and higher response rates in the 20 mg group.

## Background

Allergic rhinitis (AR) affects around 17–28.5% of the adult European population [1–3] with a considerable impact on quality of life, work and school productivity [4, 5]. The chronic nature and increasing prevalence of AR places a significant burden on patients and healthcare systems, highlighting the importance of therapeutic interventions with rapid and long-lasting effects [6].

Traditionally, AR has been classified by the duration of exposure and type of allergen into seasonal AR (SAR) or perennial AR (PAR), although newer classifications based on symptom severity and duration have emerged [7].

During SAR, the contact of the nasal mucosa with pollens leads to the degranulation and release of inflammatory mediators by mast cells and basophils which, in turn, recruit other inflammatory cells [8]. This complex allergic cascade is orchestrated by a myriad of inflammatory mediators specifically contributing to typical SAR manifestations, which can be broadly grouped into nasal (itching, sneezing, rhinorrhoea and congestion) and non-nasal (tearing, eye itching and redness). Histamine is mainly responsible for itching and sneezing symptoms [8]. Platelet-activating factor (PAF) is a key mediator of nasal congestion and rhinorrhoea responses through its involvement in vasodilatation and vascular permeability functions [9, 10].

Current guidelines recommend second-generation H1-antihistamines or intranasal corticosteroids (INCS) for AR management [11, 12]. Second-generation H1-antihistamines have been largely employed to treat SAR because of their effectiveness at reducing histamine-induced symptoms with minimal sedating effects [13] and patient’s preference for oral medications. However, the role of other inflammatory mediators as drivers of specific allergic manifestations has focused the interest on antihistamines with broader specificity. Rupatadine (Uriach and Cía, Barcelona, Spain) is a second-generation H1-antihistamine that combines a selective affinity for H1 receptor and a potent antagonism at PAF receptor [14, 15]. This unique mechanism of action allows overcoming a wide range of SAR manifestations [16, 17] with a good safety profile and minimal cross to the blood–brain barrier [18, 19].

The superior efficacy of rupatadine relative to placebo has been largely documented in several randomized controlled clinical trials (RCTs) and observational studies in patients with AR. A systematic review including 10 RCTs showed the superiority of rupatadine as compared to placebo [20], with benefits on nasal airway blockage [21], and on nasal and non-nasal symptoms [22]. Compared to active treatments, the efficacy of rupatadine was comparable to that of desloratadine [23], loratadine [24], cetirizine [25], and ebastine [26].

The clinical relevance of medications is difficult to establish in RCTs but the European Medicines Agency (EMA) guideline on the treatment of allergic rhinoconjunctivitis recommends assessing the response in terms of proportion of responders (≥ 50% reduction) [27], which has been recently used in AR studies [28].

Although previous studies clearly proved the superior efficacy of rupatadine as compared to placebo in the treatment of SAR, to date no study has evaluated the clinical relevance of rupatadine through a responder analysis and the influence of patient factors such as severity of symptoms for the two dosages of rupatadine (10 and 20 mg).

## Methods

### Study design

The main objective of this study was to evaluate the clinical relevance of rupatadine response as compared to placebo via responder analysis. To this end, we pooled data of seven randomized, double-blind studies comparing SAR response between rupatadine- and placebo-treated patients.

Since previous Ethics Committee approval was obtained for each study and no subject identifying information was disclosed, ethical approval was not required for this analysis of data. The studies were conducted in compliance with the declaration of Helsinki and its revisions, and with the ICH Guidelines on Medicinal products. All participants were previously informed and provided written informed consent.

### Study population

Major inclusion criteria included: (1) age ≥ 12 years, (2) documented history of SAR for at least 2 years, (3) positive skin prick test at inclusion or within 1 year before inclusion, and (4) a total symptom score ≥ 5 points at baseline [23–26]. Main exclusion criteria were as follows: (1) non-AR or rhinitis due to hypersensitivity to allergens other than pollen, (2) hypersensitivity to antihistamines, (3) certain concomitant conditions that may influence treatment response, namely asthma attack in the last 3 months or obstructive nasal polyps, (3) treatment with topical antihistamines in the previous 48 h, nasal decongestants in the previous 24 h, oral antihistamines, ketotifen within the previous 2 weeks, disodium cromoglycate within the previous week, systemic or topical treatment with corticosteroids, immunosuppressants, or any investigational drug within 2 weeks prior to inclusion, and (4) the presence of certain abnormal laboratory values [23–26].

### Study protocol

In brief, the studies were scheduled in three visits: Visit 1 (baseline, day 0), Visit 2 (day 7) and Visit 3 (end of study, day 14). At baseline, eligible patients were randomized to receive the active treatment (10 mg or 20 mg rupatadine) or placebo. Participants were provided with diary cards for the self-recording of symptoms twice a day (in the morning before treatment and at bedtime) and investigators instructed how to rate the severity of each symptom score. Participants reported the severity of each symptom in the patient diary before treatment initiation (day 0) and over 14 days of treatment. The investigators checked the patients’ diary cards at each follow-up visit (days 7 and 14). Since study duration was 2 weeks in all the studies except for the study of Lukat et al. (4 weeks) [23], the analysis of this study comprised 2 weeks of treatment. The characteristics of each study included in the pooled analysis are shown in Additional file 1: Table S1.

### Study intervention

Patients were administered oral rupatadine tablets at 10 mg or 20 mg or placebo once daily in the morning for 14 days. Both placebo and active treatments were administered in tablets of identical characteristics to avoid treatment identification in double-blind clinical trials [23–26]. In all comparative trials, treatments were capsulated in gelatine opaque capsules (Capsugel^®^) with the aim to preserve the double-blind conditions.

### Study outcomes

#### Symptom scores

The following symptom scores were evaluated in the pooled data at baseline and over 14 days of treatment: Total 4 Nasal Symptom Score (T4NSS), Total 2 Ocular Symptom Score (T2OSS) and Total 6 Symptom Score (T6SS).

The T4NSS is the sum of 4 individual nasal scores: rhinorrhoea, nasal congestion, nasal itching, and sneezing, each graded using a 4-point scale (0 = absent, 1 = mild, 2 = moderate, and 3 = severe), resulting in a total score of 12. The T2OSS is the sum of two ocular symptoms (eye itching and tearing), rated on a 4-point scale (0 = absent, 1 = mild, 2 = moderate, and 3 = severe), resulting in a total score of 6. The T6SS is the sum of the six symptoms of the T4NSS and T2OSS, resulting in a maximal score of 18.

#### Responder analysis

We determined the proportion of responders on days 7 and 14, defined as those patients who reached a reduction in symptom scores ≥ 50% or ≥ 75% (henceforth termed 50% or 75% response, respectively).

#### Time to response

The time to response was defined as the time to achieve a specific proportion of responders. For each response cut-off (≥ 50% or ≥ 75%), we selected the proportion of responders for whom time intervals between rupatadine groups were maximal.

#### Complete/near-to-complete response

We compared the proportion of patients reaching the complete/near-to-complete response for T4NSS and T6SS on days 7 and 14. A complete/near-to-complete response for T4NSS was defined as the combination of a T4NSS score ≤ 2 and of each individual symptom ≤ 1. To fulfil the complete/near-to-complete response for T6SS, patients required both a T6SS score ≤ 3 and of each individual symptom ≤ 1.

### Statistical analyses

Continuous variables were described by mean, standard deviation (SD) and categorical variables were described by number and percentage. Analyses were performed in the intention-to-treat population, defined as those patients who were randomized and received at least one dose of the study medication.

We used non-parametric tests since symptom scores were ordinal variables and followed a non-normal distribution. Statistical significance for symptom score evolution was determined using the non-parametric Kruskal–Wallis test followed by the Mann–Whitney test for pairwise comparisons. Differences between groups for the proportion of responders were determined with the Chi square test. Statistical analyses were performed using SAS software (SAS Institute, Cary, SC, USA) for Windows, version 9.2. The level of statistical significance was set at *p* < 0.05.

## Results

### Study population

This pooled analysis comprised data from 1470 patients: 332 treated with placebo, 662 with rupatadine 10 mg and 476 with rupatadine 20 mg. Mean age in the overall population was 33.2 years, 51.8% were female and mean duration of rupatadine treatment was 16.3 days. At baseline, mean T4NSS, T2OSS, and T6SS were statistically comparable between groups. Demographic and clinic characteristics were balanced between groups at baseline (Table 1).

**Table 1:** Clinical and demographic characteristics of the study population at baseline

	Placebo (N = 332)	Rup 10 mg (N = 662)	Rup 20 mg (N = 476)
Age (years), mean (SD)	33.0 (11.9)	32.5 (10.9)	33.2 (11.3)
Sex (women), n (%)	158 (47.6)	352 (53.2)	260 (54.6)
Weight (kg), mean (SD)	71.5 (15.4)	70.0 (14.9)	70.5 (14.9)
Height (cm), mean (SD)	169.8 (11.1)	168.9 (9.8)	168.5 (10.0)
Days of treatment, mean (SD)	17.9 (8.0)	16.7 (6.2)	14.3 (3.2)
T4NSS (0–12), mean (SD)	8.1 (1.8)	7.9 (1.8)	7.9 (1.9)
T2OSS (0–6), mean (SD)	3.2 (1.5)	2.9 (1.7)	2.8 (1.8)
T6SS (0–18), mean (SD)	11.3 (2.8)	10.8 (2.9)	10.6 (3.1)

*Rup* Rupatadine, *T4NSS* Total 4 Nasal Symptom Score, *T2OSS* Total 2 Ocular Symptom Score, *T6SS* Total 6 Symptom Score, *SD* standard deviation

### Symptom scores

**Fig. 1:**
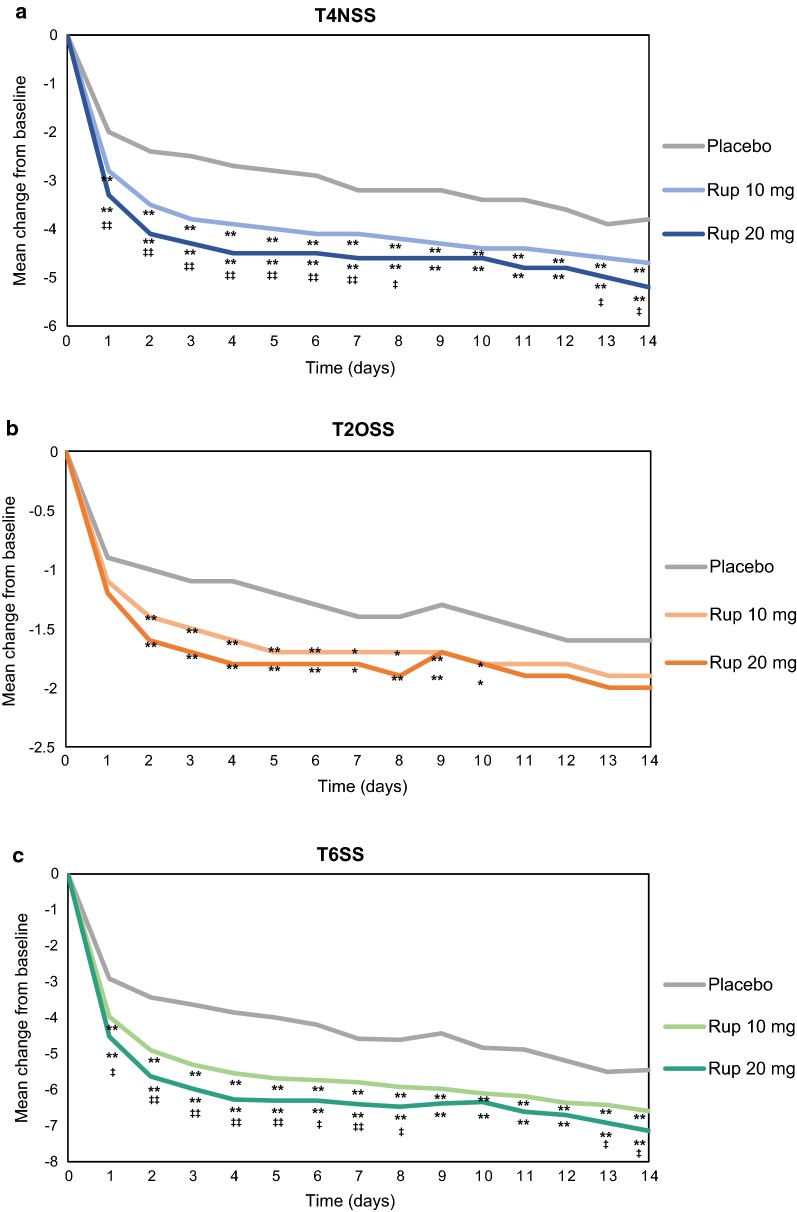
Symptom score evolution over time after treatment with rupatadine 10 mg or 20 mg for T4NSS, T2OSS, and T6SS. Data are expressed as mean change from baseline over time of treatment for: **a** T4NSS, **b** T2OSS, and **c** T6SS. Statistical significance was calculated with the Mann–Whitney test. *p < 0.05; **p < 0.01 (rupatadine groups vs placebo); ^‡^*p *< 0.05; ^‡‡^*p *< 0.01 (rupatadine 10 mg vs rupatadine 20 mg). *T4NSS* Total 4 Nasal Symptom Score, *T2OSS* Total 2 Ocular Symptom Score, *T6SS* Total 6 Symptom Score

The reduction in T4NSS was significantly higher in the active treatment groups than in the placebo group (*p *< 0.01). Among rupatadine-treated patients, those receiving 20 mg experienced higher improvements in T4NSS compared to the 10 mg group that reached statistical significance from day 1 to 8 and from day 13 to 14 (*p *< 0.05) (Fig. 1a, Additional file 1: Table S2).

The reduction in T2OSS from baseline was higher in rupatadine groups vs the placebo group, with statistically significant differences from day 2 to 10 (*p *< 0.05). The decrease from baseline was higher in the rupatadine 20 mg group than in the rupatadine 10 mg group, but differences were not statistically significant (Fig. 1b, Additional file 1: Table S2).

The change from baseline in T6SS was more pronounced in rupatadine-treated patients than in placebo-treated patients over the treatment period (*p *< 0.01). Significantly higher reductions were observed in patients treated with 20 mg compared to those treated with 10 mg from day 1 to 8 and from 13 to 14 (*p *< 0.05) (Fig. 1c, Additional file 1: Table S2).

### Responder analysis

The proportion of responders was systematically higher in rupatadine groups than in the placebo group for all the symptom scores across the two response cut-offs (≥ 50% and ≥ 75%).

**Fig. 2:**
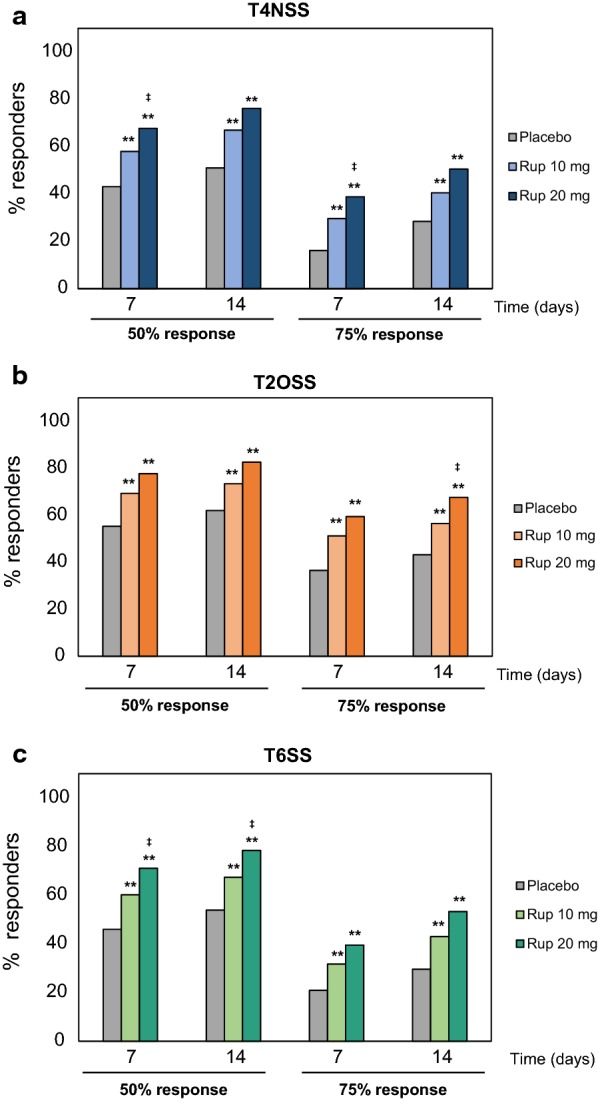
Proportion of responders for the 50% or 75% response after treatment with rupatadine 10 mg or 20 mg for T4NSS, T2OSS, and T6SS. Data are expressed as percentage of patients achieving either a 50% or 75% reduction in symptom score from baseline (50% or 75% response, respectively) for: **a** T4NSS, **b** T2OSS, and **c** T6SS. Statistical significance was calculated with the Mann–Whitney test. *p < 0.05; **p < 0.01 (rupatadine groups vs placebo); ^‡^*p *< 0.05; ^‡‡^*p *< 0.01 (rupatadine 10 mg vs rupatadine 20 mg). *T4NSS* Total 4 Nasal Symptom Score, *T2OSS* Total 2 Ocular Symptom Score, *T6SS* Total 6 Symptom Score

For the 50% reduction in T4NSS, the proportion of responders was significantly higher among rupatadine-treated patients as compared to placebo-treated patients at both visits (*p *< 0.001) and statistical significance between rupatadine groups was only observed on day 7 (*p *< 0.05). A significantly greater number of patients reached the 75% response in rupatadine groups as compared to the placebo group on days 7 and 14 (*p *< 0.001). After 7 days of treatment, the rupatadine 20 mg group attained significantly higher rates of responders compared to the 10 mg group (*p *< 0.05) (Fig. 2a).

The proportion of patients achieving the 50% response for the T2OSS was statistically higher in both rupatadine groups as compared to the placebo group (*p *< 0.001). A similar trend was observed for the 75% response, with significant differences between rupatadine groups and placebo on days 7 and 14 (*p *< 0.001), and between rupatadine 10 mg and 20 mg on day 14 (*p *< 0.05) (Fig. 2b).

A significantly higher number of patients treated with rupatadine reached the 50% response for the T6SS across the visits compared to placebo-treated patients (*p *< 0.001). Likewise, the comparison of both rupatadine doses showed statistically greater responder rates in the 20 mg group than in the 10 mg group on days 7 and 14 (*p *< 0.05). The proportion of responders for the 75% response was statistically higher in rupatadine groups as compared to the placebo group and comparable between rupatadine groups (Fig. 2c).

### Time to response

**Fig. 3:**
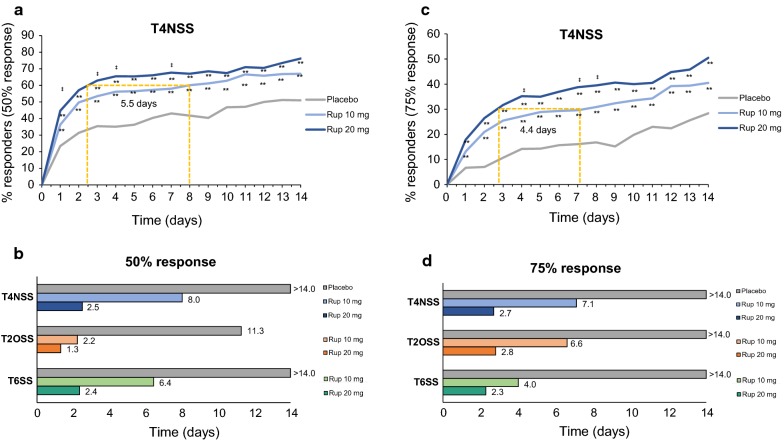
Time to achieve 50% and 75% response after treatment with rupatadine 10 mg or 20 mg for T4NSS, T2OSS, and T6SS. **a** Proportion of responders over time to achieve the 50% response in T4NSS. **c** Proportion of responders over time to achieve 75% response in T4NSS. **b** Time (days) to achieve the 50% response (T4NSS, T2OSS, and T6SS). **d** Time (days) to achieve the 75% response (T4NSS, T2OSS, and T6SS). *T4NSS* Total 4 Nasal Symptom Score, *T2OSS* Total 2 Ocular Symptom Score, *T6SS* Total 6 Symptom Score

Pooled data showed that 60% of patients reached the 50% response for the T4NSS after 2.5, 8.0 and > 14 days in the rupatadine 20 mg, rupatadine 10 mg and placebo groups, respectively (Fig. 3a, b). The time to achieve a 30% proportion of responders for the 75% response was 2.7 days in the rupatadine 20 mg group, 7.1 days in the rupatadine 10 mg group and > 14 days in the placebo group (Fig. 3c, d).

Remarkably, rupatadine groups showed narrower time intervals to achieve a percentage of responders for the T2OSS. Sixty percent of patients reached the 50% response after 1.3 days of treatment with rupatadine 20 mg, 2.2 days with rupatadine 10 mg, and 11.3 with placebo. The 75% response was achieved by 50% of patients after 2.8 days in the rupatadine 20 mg group, 6.6 days in the rupatadine 10 mg group and > 14 days in the placebo group (Fig. 3b, d).

The analysis of the T6SS showed a 60% proportion of responders after 2.4, 6.4 and > 14 days for the 50% response and a 30% proportion of responders after 2.3, 4.0 and > 14 days for the 75% response in the rupatadine 20 mg, rupatadine 10 mg and placebo groups, respectively (Fig. 3b, d).

### Complete/near-to-complete response

**Fig. 4:**
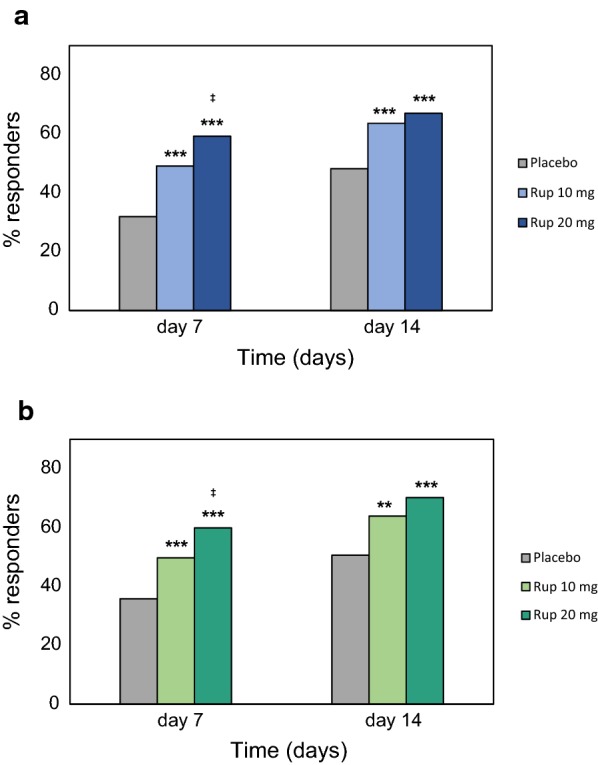
Proportion of patients achieving the complete/near-to-complete response after treatment with rupatadine 10 mg or 20 mg for T4NSS and T6SS. Data are expressed as percentage of patients achieving the complete/near-to-complete endpoints: **a** complete/near-to-complete reduction was defined as T4NSS score ≤ 2 and each symptom score ≤ 1, **b** complete/near-to-complete reduction was defined as T6SS score ≤ 3 and each symptom score ≤ 1. Statistical significance was calculated with the Mann–Whitney test. ***p < 0.001; **p < 0.01 (rupatadine groups vs placebo); ^‡^*p *< 0.05 (rupatadine 10 mg vs rupatadine 20 mg)

After 7 days of treatment, differences between rupatadine and placebo groups in the proportion of patients with a complete/near-to-complete response (T4NSS score ≤ 2 and each individual symptom score ≤ 1) were statistically significant for both pairwise comparisons (10 mg rupatadine vs placebo and 20 mg rupatadine vs placebo). The percentage of responders was statistically superior in the 20 mg group as compared to the 10 mg group on day 7 (*p *= 0.019). On day 14, a significantly higher proportion of rupatadine-treated patients achieved the complete/near-to-complete response as compared to placebo-treated patients, but responder rates were statistically similar between rupatadine groups (Fig. 4a).

A statistically higher proportion of patients reached the complete/near-to-complete response for the T6SS (T6SS score ≤ 3 and each individual symptom score ≤ 1) in the rupatadine 20 mg group as compared to the rupatadine 10 mg group and placebo group on day 7 (*p *< 0.001 and *p* = 0.019, respectively). After 14 days of treatment, a statistically higher number of patients treated with rupatadine achieved the complete/near-to-complete endpoint, but differences between rupatadine groups were not statistically significant (Fig. 4b).

## Discussion

In this responder analysis, we revealed the superior efficacy of rupatadine over placebo at improving SAR symptoms. We also evidenced that rupatadine response was dose-dependent, achieving faster and higher improvements with 20 mg over the whole follow-up period.

### Strengths and weaknesses

The main strength of this study is the comprehensive analysis performed, including data on symptom response evolution, responder proportions, and time to achieve response. To the best of our knowledge, no previous study on SAR has addressed all these analyses before, including several multicentre, double-blind RCTs with patients with moderate-severe SAR. In addition, this study analysed data from 1470 patients, representing a considerable sample size. Baseline characteristics in our study were similar to those of previous studies, reinforcing the validity of our results [23–26, 29, 30]. The main limitations of the study are the post hoc analysis and duration of the study (2 weeks), which does not allow capturing the complete stabilization of clinical symptoms. The lack of safety analyses is an additional limitation of the study. In this regard, rupatadine was safe and well-tolerated in several randomized clinical trials [17, 23, 24, 26, 31], showing a similar safety profile compared with other antihistamines such as desloratadine, loratadine, cetirizine, and ebastine [17]. Furthermore, this analysis did not include patients with mild AR who frequently self-medicate and are rarely seen by specialists. In contrast, it included patients with moderate-to-severe AR who represent the patient population treated in allergology, ear, nose and throat (ENT) or respiratory units. Additionally, the clinical relevance of these results should be interpreted considering the potential influence of patient-related factors like disease severity, presence of comorbidities or the major severity of nasal or ocular symptoms.

### Discussion of results

Upon treatment with rupatadine, symptom scores (T4NSS, T2OSS, and T6SS) gradually decreased from baseline over the 14-day treatment period, with higher improvements in the 20 mg group compared to the 10 mg group. The proportion of responders was systematically higher in rupatadine groups than in the placebo group for all the symptom scores across the two response cut-offs (≥ 50% and ≥ 75%). The time to achieve the 50% and 75% response was shorter in the rupatadine 20 mg than in the rupatadine 10 mg group and placebo groups for all the symptom scores. The proportion of patients with a complete/near-to-complete response for T4NSS and T6SS was statistically higher in rupatadine groups than in the placebo group.

One of the most important findings of this study is that, for all the analyses performed, rupatadine 20 mg provided better responses than rupatadine 10 mg or placebo, as shown by the higher reduction of symptoms and proportion of responders, and the faster onset of action. In the systematic review performed by Compalati et al., the comparison between rupatadine 10 mg and 20 mg showed no statistically significant advantage, but rupatadine 20 mg compared favourably [20].

The analysis of daily symptom scores showed that rupatadine provided both early and sustained responses, as previously described [19]. The evolution of symptom scores in patients treated with rupatadine was characterized by a rapid symptom relief followed by a gradual improvement for the following 14 days. This fast onset of action agrees with the results from pharmacokinetic and nasal challenge studies previously showing the rapid absorption of rupatadine [22, 32, 33]. In our study, the initial decrease was more abrupt from day 1 to 5, followed by more stable effects thereafter. Since nasal (T4NSS) and global (T6SS) symptoms still decreased from day 11 to 14, it would be interesting to assess rupatadine response for longer time periods to obtain the time to achieve maximal reductions. In this regard, the study of Lukat et al. confirmed the efficacy of rupatadine 10 mg after 4 weeks of treatment, with a 46% reduction of baseline symptoms [23].

In AR, establishing minimally clinical important differences that translate into clinical improvement remains controversial and has been scarcely addressed [34]. Regulatory authorities suggest measuring response to treatment using a responder analysis to demonstrate the magnitude of the clinical effect. The EMA guideline on the treatment of allergic rhinoconjunctivitis recommends the analysis in terms of responders (≥ 50% reduction) to assess the clinical relevance of treatments [27], which has been recently used in AR studies [28]. Following the EMA guideline, we found a high proportion of responders for both response cut-offs (≥ 50% and ≥ 75%), with higher rates observed at 20 mg. Remarkably, after 2 weeks of treatment with rupatadine 20 mg, a proportion of responders greater than 50% for the stringent cut-off of 75% response was observed for all the symptom scores. The high percentage of patients with a dramatic reduction in symptoms reinforces the clinical benefit of this antihistaminic treatment and can have significant consequences in clinical practice.

The EMA guideline also states that efficacy assessments should separately prove the improvement in nasal and ocular symptoms [27]. The number of patients who achieved a complete/near-to-complete response for both symptom scores was higher in rupatadine groups than in the placebo group, with higher proportions in the 20 mg group. Concretely, around 54% of patients treated with rupatadine reached both complete/near-to-complete responses after 7 days of treatment and approximately 65% (T4NSS) and 67% (T6SS) did so after 14 days of treatment.

One of the distinctive analyses of the present study is the assessment of the time to achieve a percentage of responders. Rupatadine 20 mg achieved faster a proportion of responders than rupatadine 10 mg or placebo for all symptom scores. This analysis is of great value to consider when selecting treatment dosage in patients with SAR, showing that doubling the dose of rupatadine significantly shortens the time to achieve a clinically meaningful effect (a reduction of 4.4 and 5.5 days in T4NSS for ≥ 50% and 75% responses, respectively). Rupatadine is authorized at 10 mg in most of the countries for adults and adolescents (over 12 years of age), whereas both 10 mg and 20 mg doses were recently authorized in Japan. Although 20 mg rupatadine was well-tolerated in Caucasian and Japanese patients, an increase in somnolence was observed in some patients with AR with this higher dose. Therefore, previous studies point out to a better balance between efficacy and safety at 10 mg, and justify the use of higher doses only for patients with severe symptoms [24].

We observed a differential trend between nasal and ocular symptoms. Differences between rupatadine groups in symptom reduction were not statistically significant for the T2OSS at any time point, and this symptom score clearly showed narrower time intervals to achieve a prespecified percentage of responders between rupatadine groups. These results could indicate that the resolution of ocular symptom is less dependent on rupatadine dose, which could be attributed to the oral route of administration that exerts a faster onset of action and better control on nasal symptoms [35]. However, these results should be analysed cautiously considering that the T2OSS only comprises two symptoms (eye itching and tearing) with a lesser contribution to the total score compared to the four nasal symptoms. In any case, the improvement of rupatadine on both nasal and ocular symptoms was clearly proven in the present pooled analysis.

This post hoc analysis may help physicians better assess patients’ phenotypes for treatment. Future work should comprise studies comparing several anti-H_1_ compounds according to the described criteria and correlating whether the cut-off responses defined in this study translate into quality of life improvements.

## Conclusion

This pooled analysis evaluated, for the first time, the proportion of responders upon rupatadine treatment in a large population of patients with SAR. We demonstrated the superior efficacy of rupatadine compared to placebo at improving SAR symptoms (T4NSS, T2OSS, and T6SS) over 14 days of treatment. The proportion of responders was systematically higher in rupatadine groups for both response cut-offs (50% and 75%), with significant differences favouring the 20 mg group. Shorter times were needed in rupatadine groups to achieve a specific proportion of responders. Patients treated with rupatadine 20 mg also reached higher proportions of responders for both complete/near-to-complete responses (T4NSS and T6SS).

## Supplementary information

**Additional file 1: Table S1.** Characteristics of rupatadine clinical studies included in the pooled analysis. **Table S2.** Effects of rupatadine treatment on total nasal symptom (T4NSS), ocular symptom (T2OSS), and total symptom (T6SS) scores.

## Data Availability

The datasets used and/or analyzed during the current study are available from the corresponding author on reasonable request.

## Abbreviations

AR: allergic rhinitis
EMA: European Medicines Agency
IgE: immunoglobulin E
INCS: intranasal corticosteroids
PAF: platelet-activating factor
PAR: perennial AR
RCT: Randomized Clinical Trial
SAR: seasonal AR
T2OSS: Total 2 Ocular Symptom Score
T4NSS: Total 4 Nasal Symptom Score
T6SS: Total 6 Symptom Score
