# Supplementary material for: Clinically relevant effect of rupatadine 20 mg and 10 mg in seasonal allergic rhinitis: a pooled responder analysis
Source: Clin Transl Allergy. 2019 Oct 9;9:50. doi: 10.1186/s13601-019-0293-4 (PMC6784348; doi:10.1186/s13601-019-0293-4)
Supplement: Supplementary file 1 — Additional file 1: Table S1. Characteristics of rupatadine clinical studies included in the pooled analysis. Table S2. Effects of rupatadine treatment on total nasal symptom (T4NSS), ocular symptom (T2OSS), and total symptom (T6SS) scores. [file 13601_2019_293_MOESM1_ESM.docx]

**ADDITIONAL MATERIAL**

**Table S1.** Characteristics of rupatadine clinical studies included in the pooled analysis.

| **Study** | **Design** | **Comparator** | **No. patients** | **Primary**  **endpoint** | **Symptoms** | **Duration** |
| --- | --- | --- | --- | --- | --- | --- |
| Saint-Martin *et al.* [24] | Multicentre, randomized, double-blind | RUP 10, 20mg  LOR 10mg  PL | 339 | mTDSS | Running nose  Itchy nose  Nasal congestion  Sneezing  Itchy eyes  Tearing  Itchy throat | 2 weeks |
| Guadaño *et al.* [26] | Multicentre, randomized, double-blind | RUP 10mg  EBA 10mg  PL | 250 | mTDSS | Running nose  Itchy nose  Nasal congestion  Sneezing  Itchy eyes  Tearing  Itchy throat | 2 weeks |
| Martínez-Cócera *et al*. [25] | Multicentre, randomized, double-blind | RUP 10mg  CET 10mg  PL | 249 | mTDSS | Running nose  Itchy nose  Nasal congestion  Sneezing  Itchy eyes  Tearing  Itchy throat | 2 weeks |
| Lukat *et al*. [23] | Multicentre, randomized, double-blind | RUP 10mg  DES 5mg  PL | 356 | T7SS | Running nose  Itchy nose  Nasal congestion  Sneezing  Itchy eyes  Tearing  Redness | 4 weeks* |
| RD477_21212  (data on file) | Multicentre, randomized  Double blind  Dose finding | RUP 10mg, 20mg  PL | 60 | mTDSS | Running nose  Itchy nose  Nasal congestion  Sneezing  Itchy eyes  Tearing  Itchy throat | 2 weeks |
| RD477_22115  (data on file) | Multicentre, randomized  Double blind  Dose finding | RUP 10mg, 20mg  PL | 430 | mTDSS | Running nose  Itchy nose  Nasal congestion  Sneezing  Itchy eyes  Tearing  Itchy throat | 2 weeks |
| URFC98III04  (data on file) | Multicentre, randomized  double-blind | RUP 10mg, 20mg  LOR 10mg  PL | 324 | mTDSS | Running nose  Itchy nose  Nasal congestion  Sneezing  Itchy eyes  Tearing  Itchy throat | 2 weeks |

RUP, rupatadine; DES, desloratadine; PL, placebo; LOR, loratadine; EBA, ebastine; CET, cetirizine; mDTSS, mean daily total symptom score.

* only data from the first 14 days was evaluated.

**Table S2.** Effects of rupatadine treatment on total nasal symptom (T4NSS), ocular symptom (T2OSS), and total symptom (T6SS) scores.

|  | **Placebo**  **(N = 332)** | **Rup 10mg**  **(N = 662)** | **Rup 20mg**  **(N = 476)** | **p-value^a^** |
| --- | --- | --- | --- | --- |
| **T4NSS (0-12)** |  |  |  |  |
| Day 7 |  |  |  |  |
| Mean score (SD) | 4.9 (2.9) | 3.8 (2.7)* | 3.3 (2.5)*‡ | <0.001 |
| % change^b^ (SD) | - 39.9 (57.9) | - 51.6 (48.6) | - 58.6 (30.2) |  |
| Day 14 |  |  |  |  |
| Mean score (SD) | 4.3 (3.1) | 3.3 (2.8)* | 2.6 (2.5)*‡ | <0.001 |
| % change (SD) | - 46.3 (70.5) | - 57.9 (51.9) | - 66.5 (30.2) |  |
| **T2OSS (0-6)** |  |  |  |  |
| Day 7 |  |  |  |  |
| Mean score (SD) | 1.8 (1.6) | 1.2 (1.4)* | 0.95 (1.3)* | 0.031 |
| % change (SD) | - 43.7 (8.6) | - 59.2 (14.5) | - 65.5 (29.1) |  |
| Day 14 |  |  |  |  |
| Mean score (SD) | 1.6 (1.6) | 1.0 (1.4) | 0.79 (1.2) | NS |
| % change (SD) | - 50.9 (7.9) | - 64.0 (16.4) | - 71.3 (31.8) |  |
| **T6SS (0-18)** |  |  |  |  |
| Day 7 |  |  |  |  |
| Mean score (SD) | 6.6 (4.2) | 5.0 (3.8)* | 5.0 (3.8)*‡ | <0.001 |
| % change (SD) | - 41.1 (50.4) | - 53.6 (30.5) | - 52.6 (2.6) |  |
| Day 14 |  |  |  |  |
| Mean score (SD) | 5.9 (4.4) | 4.4 (3.9)* | 3.4 (3.4)*‡ | <0.001 |
| % change (SD) | - 47.8 (60.9) | - 59.5 (31.8) | - 67.7 (2.0) |  |

NS, not significant; Rup, Rupatadine; T4NSS = Total 4 Nasal Symptom Score; T2OSS = Total 2 Ocular Symptom Score; T6SS = Total 6 Symptom Score; SD = standard deviation.

^a^ Statistical significance was determined using the non-parametric Kruskal-Wallis test.

^b^% change from baseline

* indicates *p* < 0.05 (rupatadine groups vs placebo) calculated with the Mann-Whitney test.

‡ indicates *p* < 0.05 (rupatadine 10 mg vs rupatadine 20 mg) calculated with the Mann-Whitney test.
